# 1-(2-Hy­droxy­benzo­yl)thio­semicarbazide hemihydrate

**DOI:** 10.1107/S1600536810044259

**Published:** 2010-11-06

**Authors:** Ming-zhi Song, Chuangang Fan

**Affiliations:** a College of Chemistry and Chemical Technology, Binzhou University, Binzhou 256600, Shandong, People’s Republic of China

## Abstract

The asymmetric unit of the title compound, C_8_H_9_N_3_O_2_S·0.5H_2_O, contains two thiosemicarbazide mol­ecules with the short distance of 3.521 (3) Å between the centroids of the benzene rings, and one water mol­ecule. In the two independent mol­ecules, the benzene rings and the thio­semicarbazone fragments are twisted at 9.2 (3) and 18.5 (3)°. An extensive three-dimensional hydrogen-bonding network, formed by inter­molecular N—H⋯O, N—H⋯S and O—H⋯O hydrogen bonds, consolidates the crystal packing.

## Related literature

For the biological activities of thio­semicarbazide derivatives, see: Desai *et al.* (1984 ▶); Shukla *et al.* (1984 ▶). For related structures, see: Gors *et al.* (1979 ▶); Jin (2007 ▶).
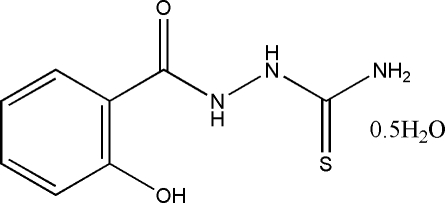


## Experimental

### 

#### Crystal data

C_8_H_9_N_3_O_2_S·0.5H_2_O
*M* *_r_* = 440.50
Monoclinic, 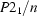

*a* = 9.0718 (11) Å
*b* = 21.608 (2) Å
*c* = 10.1035 (13) Å
β = 90.173 (1)°
*V* = 1980.5 (4) Å^3^
*Z* = 4
Mo *K*α radiation
μ = 0.31 mm^−1^
*T* = 298 K
0.45 × 0.20 × 0.17 mm

#### Data collection

Bruker SMART APEX CCD area-detector diffractometer
Absorption correction: multi-scan (*SADABS*; Sheldrick, 1996 ▶) *T*_min_ = 0.873, *T*_max_ = 0.949
9862 measured reflections
3487 independent reflections
2202 reflections with *I* > 2σ(*I*)
*R*_int_ = 0.041

#### Refinement

*R*[*F* ^2^ > 2σ(*F* ^2^)] = 0.047
*wR*(*F* ^2^) = 0.122
*S* = 1.01
3487 reflections
262 parameters
H-atom parameters constrained
Δρ_max_ = 0.37 e Å^−3^
Δρ_min_ = −0.33 e Å^−3^

### 

Data collection: *SMART* (Bruker, 2007 ▶); cell refinement: *SAINT* (Bruker, 2007 ▶); data reduction: *SAINT*; program(s) used to solve structure: *SHELXS97* (Sheldrick, 2008 ▶); program(s) used to refine structure: *SHELXL97* (Sheldrick, 2008 ▶); molecular graphics: *SHELXTL* (Sheldrick, 2008 ▶); software used to prepare material for publication: *SHELXTL*.

## Supplementary Material

Crystal structure: contains datablocks I, global. DOI: 10.1107/S1600536810044259/cv2777sup1.cif

Structure factors: contains datablocks I. DOI: 10.1107/S1600536810044259/cv2777Isup2.hkl

Additional supplementary materials:  crystallographic information; 3D view; checkCIF report

## Figures and Tables

**Table 1:** Hydrogen-bond geometry (Å, °)

*D*—H⋯*A*	*D*—H	H⋯*A*	*D*⋯*A*	*D*—H⋯*A*
N2—H2⋯S1^i^	0.86	2.58	3.361 (3)	151
N5—H5⋯S1^ii^	0.86	2.83	3.404 (3)	125
N6—H6*A*⋯O2^iii^	0.86	2.16	2.984 (3)	160
N6—H6*B*⋯S2^iv^	0.86	2.85	3.647 (3)	156
O4—H4⋯O1^ii^	0.82	1.93	2.724 (3)	162
O5—H5*B*⋯O5^v^	0.85	1.95	2.728 (10)	152
N3—H3*A*⋯O3	0.86	2.12	2.918 (3)	154
N3—H3*B*⋯O5	0.86	2.30	3.110 (5)	156
N5—H5⋯O4	0.86	1.99	2.629 (3)	130
O2—H2*A*⋯O1	0.82	1.83	2.552 (3)	146
O5—H5*D*⋯S1	0.85	2.66	3.398 (4)	147

Symmetry codes: (i) 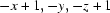; (ii) 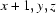; (iii) 
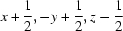
; (iv) 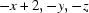; (v) 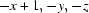.

**Fig. 1.**
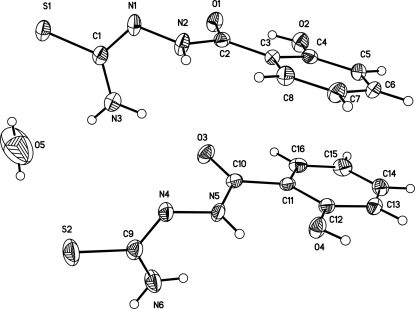
The content of asymmetric unit of the title compound showing the atomic numbering scheme and 30% probability displacement ellipsoids.

## supplementary crystallographic information

### Comment

Thiosemicarbazide compounds exhibit various biological activities such as anti-bacterial, anti-fungal and especially anti-tuberculosis (Shukla *et al.*, 1984; Desai *et al.*, 1984). Herewith we present the title compound (I), a new thiosemicarbazide compound.

The asymmetric unit of (I) contains two independent molecules and one crystalline water molecule (Fig. 1). The bond lengths and angles are normal and comparable to the values observed in similar compounds (Gors *et al.*, 1979; Jin, 2007). In two independent molecules, the benzene rings and the thiosemicarbazone fragments (C/N/N) are twisted at 9.2 (3)° and 18.5 (3)°, respectively.

In the crystal structure, the three-dimensional network structure was formed by the intermolecular N—H···O, N—H···S and O—H···O hydrogen bonds (Table 1).

### Experimental

Salicylyl hydrazine (10 mmol), potassium thiocyanate (12 mmol) and 10 ml me thanol-water (1:1) were mixed in 50 ml flask. After refluxing 12 h at 373 K, the resulting mixture was recrystalized from solution, affording the title compound as a colorless crystalline solid.

### Refinement

All H atoms were placed in geometrically idealized positions (N—H = 0.86, O—H= 0.82-0.85 and C—H = 0.93 Å) and treated as riding on their parent atoms, with *U*_iso_(H) = 1.2U-1.5 U_eq_ of the parent atom.

### Crystal data

**Table:**

C_16_H_20_N_6_O_5_S_2_	*F*(000) = 916
*M**_r_* = 440.50	*D*_x_ = 1.474 Mg m^−^^3^
Monoclinic, *P*2_1_/*n*	Mo *K*α radiation, λ = 0.71073 Å
*a* = 9.0718 (11) Å	Cell parameters from 2367 reflections
*b* = 21.608 (2) Å	θ = 2.2–25.7°
*c* = 10.1035 (13) Å	µ = 0.31 mm^−^^1^
β = 90.173 (1)°	*T* = 298 K
*V* = 1980.5 (4) Å^3^	Block, yellow
*Z* = 4	0.45 × 0.20 × 0.17 mm

### Data collection

**Table:**

Bruker SMART APEX CCD area-detector diffractometer	3487 independent reflections
Radiation source: fine-focus sealed tube	2202 reflections with *I* > 2σ(*I*)
graphite	*R*_int_ = 0.041
φ and ω scans	θ_max_ = 25.0°, θ_min_ = 1.9°
Absorption correction: multi-scan (*SADABS*; Sheldrick, 1996)	*h* = −10→10
*T*_min_ = 0.873, *T*_max_ = 0.949	*k* = −21→25
9862 measured reflections	*l* = −11→12

### Refinement

**Table:**

Refinement on *F*^2^	Primary atom site location: structure-invariant direct methods
Least-squares matrix: full	Secondary atom site location: difference Fourier map
*R*[*F*^2^ > 2σ(*F*^2^)] = 0.047	Hydrogen site location: inferred from neighbouring sites
*wR*(*F*^2^) = 0.122	H-atom parameters constrained
*S* = 1.01	*w* = 1/[σ^2^(*F*_o_^2^) + (0.0468*P*)^2^ + 1.2463*P*] where *P* = (*F*_o_^2^ + 2*F*_c_^2^)/3
3487 reflections	(Δ/σ)_max_ < 0.001
262 parameters	Δρ_max_ = 0.37 e Å^−^^3^
0 restraints	Δρ_min_ = −0.32 e Å^−^^3^

### Special details

**Table:**

Geometry. All e.s.d.'s (except the e.s.d. in the dihedral angle between two l.s. planes) are estimated using the full covariance matrix. The cell e.s.d.'s are taken into account individually in the estimation of e.s.d.'s in distances, angles and torsion angles; correlations between e.s.d.'s in cell parameters are only used when they are defined by crystal symmetry. An approximate (isotropic) treatment of cell e.s.d.'s is used for estimating e.s.d.'s involving l.s. planes.
Refinement. Refinement of *F*^2^ against ALL reflections. The weighted *R*-factor *wR* and goodness of fit *S* are based on *F*^2^, conventional *R*-factors *R* are based on *F*, with *F* set to zero for negative *F*^2^. The threshold expression of *F*^2^ > σ(*F*^2^) is used only for calculating *R*-factors(gt) *etc*. and is not relevant to the choice of reflections for refinement. *R*-factors based on *F*^2^ are statistically about twice as large as those based on *F*, and *R*- factors based on ALL data will be even larger.

### Fractional atomic coordinates and isotropic or equivalent isotropic displacement parameters (Å^2^)

**Table:**

	*x*	*y*	*z*	*U*_iso_*/*U*_eq_	Occ. (<1)
N1	0.4021 (3)	0.07227 (12)	0.5682 (3)	0.0401 (7)	
H1	0.3330	0.0634	0.6231	0.048*	
N2	0.5273 (3)	0.10117 (11)	0.6152 (3)	0.0392 (7)	
H2	0.6033	0.0794	0.6354	0.047*	
N3	0.4849 (3)	0.07466 (12)	0.3544 (3)	0.0443 (7)	
H3A	0.5612	0.0950	0.3806	0.053*	
H3B	0.4746	0.0655	0.2721	0.053*	
N4	0.8800 (3)	0.06010 (11)	0.3372 (3)	0.0399 (7)	
H4'	0.8275	0.0385	0.3908	0.048*	
N5	0.9398 (3)	0.11556 (11)	0.3803 (3)	0.0362 (7)	
H5	1.0322	0.1177	0.3992	0.043*	
N6	0.9674 (3)	0.07614 (13)	0.1292 (3)	0.0527 (8)	
H6A	0.9949	0.1125	0.1534	0.063*	
H6B	0.9827	0.0639	0.0495	0.063*	
O1	0.4251 (2)	0.19444 (10)	0.5943 (2)	0.0386 (6)	
O2	0.5712 (3)	0.29296 (10)	0.6415 (2)	0.0441 (6)	
H2A	0.5042	0.2722	0.6100	0.066*	
O3	0.7237 (2)	0.16510 (10)	0.3629 (2)	0.0439 (6)	
O4	1.1396 (2)	0.17295 (9)	0.5240 (2)	0.0408 (6)	
H4	1.2180	0.1806	0.5612	0.061*	
O5	0.3935 (7)	0.0087 (2)	0.0932 (4)	0.172 (2)	
H5D	0.3575	−0.0063	0.1638	0.206*	0.50
H5B	0.4780	0.0030	0.0591	0.206*	0.50
S1	0.23175 (9)	0.01686 (4)	0.39559 (9)	0.0415 (3)	
S2	0.84286 (14)	−0.03260 (5)	0.17448 (11)	0.0703 (4)	
C1	0.3839 (4)	0.05757 (13)	0.4406 (3)	0.0336 (8)	
C2	0.5320 (3)	0.16295 (14)	0.6297 (3)	0.0307 (7)	
C3	0.6647 (3)	0.19040 (13)	0.6870 (3)	0.0287 (7)	
C4	0.6763 (3)	0.25507 (14)	0.6910 (3)	0.0315 (7)	
C5	0.7998 (4)	0.28273 (16)	0.7474 (3)	0.0404 (8)	
H5A	0.8066	0.3256	0.7513	0.049*	
C6	0.9113 (4)	0.24687 (18)	0.7971 (3)	0.0448 (9)	
H6	0.9938	0.2657	0.8342	0.054*	
C7	0.9031 (4)	0.18322 (17)	0.7929 (3)	0.0445 (9)	
H7	0.9797	0.1593	0.8267	0.053*	
C8	0.7813 (3)	0.15549 (15)	0.7387 (3)	0.0377 (8)	
H8	0.7761	0.1125	0.7361	0.045*	
C9	0.9021 (4)	0.03945 (15)	0.2138 (4)	0.0412 (8)	
C10	0.8546 (3)	0.16613 (14)	0.3929 (3)	0.0307 (7)	
C11	0.9288 (3)	0.22405 (13)	0.4381 (3)	0.0288 (7)	
C12	1.0660 (3)	0.22665 (13)	0.5004 (3)	0.0303 (7)	
C13	1.1241 (4)	0.28340 (14)	0.5386 (3)	0.0370 (8)	
H13	1.2142	0.2849	0.5826	0.044*	
C14	1.0501 (4)	0.33711 (15)	0.5122 (3)	0.0425 (9)	
H14	1.0910	0.3749	0.5365	0.051*	
C15	0.9147 (4)	0.33536 (15)	0.4495 (3)	0.0431 (9)	
H15	0.8642	0.3719	0.4314	0.052*	
C16	0.8552 (4)	0.27936 (14)	0.4143 (3)	0.0373 (8)	
H16	0.7633	0.2784	0.3733	0.045*	

### Atomic displacement parameters (Å^2^)

**Table:**

	*U*^11^	*U*^22^	*U*^33^	*U*^12^	*U*^13^	*U*^23^
N1	0.0370 (17)	0.0357 (16)	0.0475 (18)	−0.0102 (13)	−0.0024 (14)	−0.0063 (14)
N2	0.0284 (16)	0.0278 (15)	0.0613 (19)	−0.0024 (12)	−0.0136 (13)	−0.0030 (13)
N3	0.0396 (18)	0.0449 (17)	0.0485 (18)	−0.0078 (14)	−0.0004 (15)	−0.0042 (14)
N4	0.0388 (17)	0.0296 (15)	0.0514 (18)	−0.0092 (12)	0.0036 (14)	−0.0057 (13)
N5	0.0256 (15)	0.0272 (14)	0.0558 (18)	−0.0054 (12)	−0.0035 (13)	−0.0093 (13)
N6	0.078 (2)	0.0351 (17)	0.0453 (18)	−0.0171 (16)	0.0066 (16)	−0.0064 (15)
O1	0.0281 (13)	0.0323 (12)	0.0555 (14)	0.0039 (10)	−0.0091 (11)	−0.0004 (11)
O2	0.0432 (15)	0.0294 (12)	0.0596 (15)	0.0017 (10)	−0.0031 (12)	−0.0032 (11)
O3	0.0241 (13)	0.0436 (14)	0.0639 (16)	−0.0005 (10)	−0.0073 (11)	−0.0033 (12)
O4	0.0315 (13)	0.0331 (13)	0.0577 (15)	0.0027 (10)	−0.0120 (11)	−0.0027 (11)
O5	0.299 (7)	0.114 (3)	0.102 (3)	−0.017 (4)	0.030 (4)	−0.006 (3)
S1	0.0350 (5)	0.0327 (5)	0.0567 (6)	−0.0036 (4)	−0.0089 (4)	−0.0056 (4)
S2	0.1012 (9)	0.0420 (6)	0.0677 (7)	−0.0309 (6)	0.0138 (6)	−0.0172 (5)
C1	0.0309 (19)	0.0198 (16)	0.050 (2)	0.0055 (13)	−0.0033 (16)	−0.0016 (15)
C2	0.0303 (18)	0.0316 (18)	0.0302 (17)	−0.0006 (15)	0.0035 (14)	0.0010 (14)
C3	0.0264 (17)	0.0303 (17)	0.0293 (17)	−0.0022 (13)	0.0012 (13)	−0.0030 (14)
C4	0.0308 (19)	0.0325 (18)	0.0313 (18)	0.0019 (14)	0.0051 (14)	−0.0033 (15)
C5	0.041 (2)	0.0376 (19)	0.043 (2)	−0.0130 (16)	0.0075 (17)	−0.0115 (16)
C6	0.030 (2)	0.064 (3)	0.040 (2)	−0.0169 (18)	0.0041 (16)	−0.0147 (19)
C7	0.030 (2)	0.058 (2)	0.045 (2)	−0.0004 (17)	−0.0064 (16)	−0.0021 (18)
C8	0.036 (2)	0.0357 (19)	0.0410 (19)	0.0022 (15)	−0.0036 (16)	−0.0021 (16)
C9	0.040 (2)	0.0313 (19)	0.052 (2)	−0.0035 (15)	−0.0020 (17)	−0.0036 (17)
C10	0.0270 (19)	0.0315 (18)	0.0337 (18)	−0.0003 (14)	0.0046 (14)	0.0012 (14)
C11	0.0253 (17)	0.0284 (17)	0.0328 (17)	−0.0003 (13)	0.0068 (14)	−0.0011 (14)
C12	0.0288 (18)	0.0285 (17)	0.0338 (18)	0.0000 (14)	0.0034 (14)	−0.0009 (14)
C13	0.0326 (19)	0.0354 (19)	0.043 (2)	−0.0050 (15)	0.0012 (16)	−0.0043 (16)
C14	0.048 (2)	0.0285 (19)	0.051 (2)	−0.0053 (17)	0.0093 (18)	−0.0096 (16)
C15	0.046 (2)	0.0274 (18)	0.056 (2)	0.0080 (16)	0.0064 (18)	0.0025 (16)
C16	0.034 (2)	0.0360 (19)	0.042 (2)	0.0057 (15)	0.0076 (15)	0.0017 (16)

### Geometric parameters (Å, °)

**Table:**

N1—C1	1.338 (4)	S1—C1	1.697 (3)
N1—N2	1.379 (3)	S2—C9	1.694 (3)
N1—H1	0.8600	C2—C3	1.460 (4)
N2—C2	1.344 (4)	C3—C8	1.399 (4)
N2—H2	0.8600	C3—C4	1.402 (4)
N3—C1	1.319 (4)	C4—C5	1.391 (4)
N3—H3A	0.8600	C5—C6	1.369 (5)
N3—H3B	0.8600	C5—H5A	0.9300
N4—C9	1.340 (4)	C6—C7	1.378 (5)
N4—N5	1.385 (3)	C6—H6	0.9300
N4—H4'	0.8600	C7—C8	1.370 (4)
N5—C10	1.345 (4)	C7—H7	0.9300
N5—H5	0.8600	C8—H8	0.9300
N6—C9	1.308 (4)	C10—C11	1.492 (4)
N6—H6A	0.8600	C11—C16	1.390 (4)
N6—H6B	0.8600	C11—C12	1.394 (4)
O1—C2	1.237 (3)	C12—C13	1.389 (4)
O2—C4	1.351 (4)	C13—C14	1.367 (4)
O2—H2A	0.8200	C13—H13	0.9300
O3—C10	1.225 (3)	C14—C15	1.381 (5)
O4—C12	1.359 (3)	C14—H14	0.9300
O4—H4	0.8200	C15—C16	1.371 (4)
O5—H5D	0.8496	C15—H15	0.9300
O5—H5B	0.8500	C16—H16	0.9300
			
C1—N1—N2	122.6 (3)	C4—C5—H5A	120.0
C1—N1—H1	118.7	C5—C6—C7	120.9 (3)
N2—N1—H1	118.7	C5—C6—H6	119.5
C2—N2—N1	120.9 (3)	C7—C6—H6	119.5
C2—N2—H2	119.5	C8—C7—C6	119.5 (3)
N1—N2—H2	119.5	C8—C7—H7	120.3
C1—N3—H3A	120.0	C6—C7—H7	120.3
C1—N3—H3B	120.0	C7—C8—C3	121.4 (3)
H3A—N3—H3B	120.0	C7—C8—H8	119.3
C9—N4—N5	121.4 (3)	C3—C8—H8	119.3
C9—N4—H4'	119.3	N6—C9—N4	118.4 (3)
N5—N4—H4'	119.3	N6—C9—S2	123.2 (3)
C10—N5—N4	120.5 (3)	N4—C9—S2	118.4 (3)
C10—N5—H5	119.7	O3—C10—N5	121.3 (3)
N4—N5—H5	119.7	O3—C10—C11	121.8 (3)
C9—N6—H6A	120.0	N5—C10—C11	116.8 (3)
C9—N6—H6B	120.0	C16—C11—C12	118.1 (3)
H6A—N6—H6B	120.0	C16—C11—C10	116.9 (3)
C4—O2—H2A	109.5	C12—C11—C10	125.0 (3)
C12—O4—H4	109.5	O4—C12—C13	121.3 (3)
H5D—O5—H5B	129.4	O4—C12—C11	118.8 (3)
N3—C1—N1	119.1 (3)	C13—C12—C11	119.9 (3)
N3—C1—S1	122.3 (3)	C14—C13—C12	120.6 (3)
N1—C1—S1	118.6 (3)	C14—C13—H13	119.7
O1—C2—N2	119.4 (3)	C12—C13—H13	119.7
O1—C2—C3	122.4 (3)	C13—C14—C15	120.2 (3)
N2—C2—C3	118.2 (3)	C13—C14—H14	119.9
C8—C3—C4	118.1 (3)	C15—C14—H14	119.9
C8—C3—C2	123.4 (3)	C16—C15—C14	119.5 (3)
C4—C3—C2	118.5 (3)	C16—C15—H15	120.3
O2—C4—C5	117.2 (3)	C14—C15—H15	120.3
O2—C4—C3	122.7 (3)	C15—C16—C11	121.7 (3)
C5—C4—C3	120.0 (3)	C15—C16—H16	119.2
C6—C5—C4	120.1 (3)	C11—C16—H16	119.2
C6—C5—H5A	120.0		

### Hydrogen-bond geometry (Å, °)

**Table:**

*D*—H···*A*	*D*—H	H···*A*	*D*···*A*	*D*—H···*A*
N2—H2···S1^i^	0.86	2.58	3.361 (3)	151
N5—H5···S1^ii^	0.86	2.83	3.404 (3)	125
N6—H6A···O2^iii^	0.86	2.16	2.984 (3)	160
N6—H6B···S2^iv^	0.86	2.85	3.647 (3)	156
O4—H4···O1^ii^	0.82	1.93	2.724 (3)	162
O5—H5B···O5^v^	0.85	1.95	2.728 (10)	152
N3—H3A···O3	0.86	2.12	2.918 (3)	154
N3—H3B···O5	0.86	2.30	3.110 (5)	156
N5—H5···O4	0.86	1.99	2.629 (3)	130
O2—H2A···O1	0.82	1.83	2.552 (3)	146
O5—H5D···S1	0.85	2.66	3.398 (4)	147

Symmetry codes: (i) −*x*+1, −*y*, −*z*+1; (ii) *x*+1, *y*, *z*; (iii) *x*+1/2, −*y*+1/2, *z*−1/2; (iv) −*x*+2, −*y*, −*z*; (v) −*x*+1, −*y*, −*z*.
